# Expansion of stereotactic work envelope using transformation matrices and geometric algebra for neurosurgery

**DOI:** 10.1007/s13534-024-00434-8

**Published:** 2024-11-05

**Authors:** Basel Sharaf, Seth Lewis, David Choung, Abhinav Goyal, Kristen M. Scheitler, Lydia S. Hong, Charles D. Blaha, Barbara Hanna, Kyungwon Chang, Jason Yuen, Yoonbae Oh, Hojin Shin, Sanjeet Grewal, Jin Woo Chang, Kai Miller, Kendall H. Lee

**Affiliations:** 1 Division of Plastic Surgery, Mayo Clinic, Rochester, MN USA; 2 NaviNetics, Inc, Rochester, MN USA; 3 Neural Engineering Laboratories, Mayo Clinic, Rochester, MN USA; 4 Department of Biomedical Engineering, Mayo Clinic, Rochester, MN USA; 5 Department of Neurosurgery, Mayo Clinic, Rochester, MN USA; 6 Department of Neurological Surgery, Mayo Clinic, Jacksonville, FL USA; 7 Department of Neurosurgery, Samsung Medical Center, Seoul, South Korea; 8 Department of Neurosurgery, Korea University Anam Hospital, Seoul, South Korea; 9 Department of Neurosurgery, Yonsei University College of Medicine, Seoul, South Korea

**Keywords:** Stereotactic frame, Geometric algebra, Deep brain stimulation, Neurosurgical navigation

## Abstract

Stereotactic systems have traditionally used Cartesian coordinate combined with linear algebraic mathematical models to navigate the brain. Previously, the development of a novel stereotactic system allowed for improved patient comfort, reduced size, and carried through a simplified interface for surgeons. The system was designed with a work envelope and trajectory range optimized for deep brain stimulation applications only. However, it could be applied in multiple realms of neurosurgery by spanning the entire brain. To this end, a system of translational and rotational adapters was developed to allow total brain navigation capabilities. Adapters were designed to fit onto a Skull Anchor Key of a stereotactic frame system to allow for rotation and translation of the work envelope. Mathematical formulas for the rotations and translations associated with each adapter were developed. Mechanical and image-guided accuracies were examined using a ground truth imaging phantom. The system’s clinical workflow and its ability to reliably and accurately be used in a surgical scenario were investigated using a cadaver head and computed tomography guidance. Eight adapters designed and 3D-printed allowed the work envelope to be expanded to the entire head. The mechanical error was 1.75 ± 0.09 mm (*n* = 20 targets), and the cadaver surgical targeting error was 1.18 ± 0.28 mm (*n* = 10 implantations). The novel application of conventional and geometric algebra in conjunction with hardware modifications significantly expands the work envelope of the stereotactic system to the entire cranial cavity. This approach greatly extends the clinical applications by the system.

## Introduction

In neuromodulation, accurate targeting to navigate the brain is essential, notably for implantation of deep brain stimulation (DBS) electrodes [1, 2]. Stereotactic surgery was introduced in the early 1900s to achieve highly accurate targeting during surgical operations and was soon adapted into neurosurgery to target specific points within the brain [3–5]. Traditional stereotactic neurosurgery has been time tested to be safe and accurate, but these benefits come at the cost of the inherent bulk and cumbersome design of present commercially available frame-based systems, sacrificing patient comfort [6–9]. In addition, many of these frame-based systems have a work envelope limited to the anterior and posterior quadrants of the cranial cavity to target standard DBS brain regions such as the GPi and STN. However, there is need for a more versatile and comfortable system with an expanded work envelope to enable additional stereotactic neurosurgical operations to be performed.

To improve patient comfort and expand the work envelope of stereotactic neurosurgery, the Mayo Neural Engineering and Precision Surgery Lab has recently developed a novel stereotactic frame system [10] that combines the advantages of existing frame-based systems while improving patient comfort. In pre-clinical testing, the stereotactic system was tested to be on par with existing frame-based systems used clinically [10, 11]. Although this system was originally designed with a fixed work envelope and a trajectory range optimized for DBS applications, it is desirable to expand the work envelope and trajectory of this system from central deep targets and superior-to-inferior trajectories to allow for total brain navigation capabilities. This would enable expanding the system’s stereotactic capabilities to a wider range of targets that could be applied to diagnose and treat neurological diseases and disorders beyond the indications of DBS [12–16]. To achieve this work envelope expansion, a system of translational and rotational adapters has been developed using non-conventional mathematical geometry.

Traditional stereotactic neurosurgery has been performed by using stereotactic systems that use the Cartesian coordinate system and linear algebraic mathematical models to navigate the brain [17–20]. With the development of rotational adapters the work envelope can be expanded to the entire cranial cavity. This is possible by employing the principles of geometric algebra, a system that yields transformations which are less cumbersome than linear algebraic mathematical models. The Cartesian system of rotating vectors using 3-dimensional rotation matrices is rather arduous to visualize and describe due to large matrices that contain products of multiple trigonometric functions. On the other hand, geometric algebra (also known as the Clifford algebra) [21], is a branch of mathematics that incorporates the usage of standard vector algebra along with a few additional constructs to form a system for describing rotations, which subsumes other rotation describing systems such as Euler angles and quaternions [21, 22]. Rather than rotations being described in terms of an axis of rotation, geometric algebra describes rotations in terms of a plane of rotation by using scalars, vectors, bivectors, and a trivector. Using this mathematical system to describe the work envelope expansions achieved by the adapters should make it possible to extend the operational and clinical applications of this stereotactic system.

The purpose of this study was to develop a series of translational and rotational adapters that work with the new stereotactic frame and to describe the associated coordinate system changes using the principles of geometric algebra to allow for total brain navigation capabilities. The first objective of this study was to design and build a set of adapters that interface with the “Skull Anchor Key” of the frame system, allowing for both rotation and translation of the work envelope for targeting different brain quadrants. The second objective was to develop the mathematical formulas for the transformations achieved by each adapter using both traditional transformation matrices and geometric algebra. The third objective was to test the operational mechanics and examine the system’s mechanical and image-guided accuracies. Lastly, the fourth objective was to methodically assess each adapter “Skull Anchor Key” in a mock cadaveric surgical scenario to confirm the reliability and accuracy to target various brain sites with the new stereotactic frame.

## Methods

**Fig. 1:**
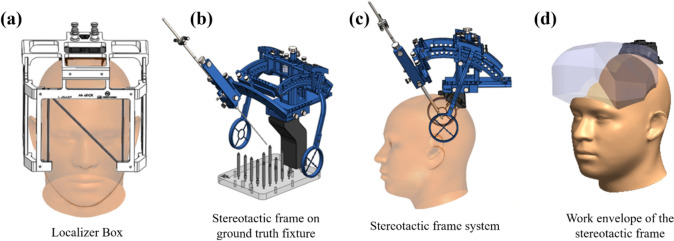
Design of frame and localizer. **a** 3-Dimensional image of CT localizer box. **b** 3-Dimensional image of the frame on the image calibration fixture that was used to test the mechanical accuracy of the frame. **c** 3-Dimensional image of the frame that is currently being used in real world applications, especially in deep brain stimulation surgeries. **d** Work envelope of the stereotactic frame

A total of eight adapters were initially designed and fabricated by 3D-printing for full cranial cavity coverage. Mechanical accuracy and phantom testing were performed with all eight adapters. Mock-surgical testing was performed with three adapters using one cadaveric specimen. Figure 1a depicts an N-bar localizer mounted on a Skull Anchor Key. Figure 1b shows the frame mounted on a ground truth fixture to test mechanical accuracy. Figure 1c illustrates the frame mounted on a human cranium. Figure 1d shows the work envelope of the stereotactic frame with the proposed adapters. The stereotactic frame followed the same principles as other arc-centered frame-based stereotactic systems [10].

### Adapter design

The stereotactic system included the new targeting device (frame) and the computed tomography (CT) N-bar or magnetic resonance (MR) imaging localizers that were attached to a small skull-mounted device platform called the “Skull Anchor Key” [10]. To develop the work envelope expanding adapters for the “Skull Anchor Key,” several design criteria were considered. These criteria included the ability to have both rotational and translational change capability, fit to the existing key and be re-attachable. The design of the adapter was performed with the aid of Solidworks™ (Dassault Systèmes™, France). After design, 3D-printing was conducted with Ultimaker™ S5 3D Printers (Ultimaker™, Netherlands) using tough polylactic acid. Tough polylactic acid was chosen due to its stability as functional prototype that minimized delamination and warping. Each adapter was capable of varying translations and rotations about all six degrees of freedom as seen in Table 1.

**Table 1:** Changes made to the original Cartesian coordinate plane in each of the six degrees of freedom by all adapters

Adapters	Translation (mm)			Rotation (°)		
	X	Y	Z	X_0_	Y_0_	Z_0_
1	− 200	− 87.3	− 3.7	40	0	180
2	− 200	− 57.1	− 3.4	60	0	180
3	− 205	36.9	− 80.3	32	0	270
4	5	− 132.8	25.7	32	0	90
5	− 243.7	− 60.6	− 25.1	40	0	210
6	− 129.5	− 137.2	39.2	40	0	150
7	− 243.6	− 51	− 33.1	40	0	215
8	− 120.2	− 138.8	40.6	40	0	145

The Z and X angle rotations allowed for optimized placement of the work envelope with each adapter

### Localizers

The CT localizer utilized N-Bar fiducials and has two side plates and one anterior plate. The side plates were positioned 190 mm laterally from each other while the midpoint of the anterior plate was exactly 110 mm from the focus of the four parallel rods. For visualization in CT, the rods were made of carbon fiber and with a diameter of 2 mm. The parallel bars of the ‘N’ in each plate were positioned 120 mm apart, and the diagonal rod connecting the parallel rods was at a 45° angle. This design allowed for existing surgical software to be used for target planning.

### Mechanical accuracy testing

To assess the mechanical accuracy of the adapter system, an acrylic imaging phantom was built in-house that contained 25 points of known coordinates that are accurate to 1/1000 of an inch (25.4 μm). These points, in reference to the targeting device, lie at locations providing a significant expansion to the previous work envelope. The frame was mounted to the testing device along with each respective adapter, and a 150 mm targeting probe was secured to the targeting device delivery platform. The frame was adjusted to target multiple phantom points with the tip of the probe, and the frame X, Y, and Z coordinate readouts were compared against the true coordinates of the point to determine the 3D Euclidean error. 3D distances were calculated using the 3D Euclidean distance equation:

$$D= \sqrt{{{(x}_{2}-{x}_{1})}^{2}+{{(y}_{2}-{y}_{1})}^{2}+{{(z}_{2}-{z}_{1})}^{2}}$$

For each variable in the equation above, subscripts 1 and 2 represent observed and expected coordinates, respectively. The process of targeting test points was repeated by two independent examiners to account for inter-user variance. All data are presented as mean ± standard error of the mean (SEM).

### Human cadaver testing

A CT-guided mock-DBS surgical procedure using the 3D-printed adapters with the stereotactic system was developed and performed on a human cadaver and deemed exempt by the Mayo Clinic’s Institutional Review Board (Supplemental Information). The specimen group consisted of one male human cadaver head and the CT imaging was conducted in a Siemens™ Somatom Definition Flash scanner (Slice thickness 5 mm, Rotations time 1 s, 120 kV, CTDI 107 mGy, FOV 300 mm). The device platform was secured to the cadaveric specimen for a CT scan with the localizer box to establish the reference coordinate system for the brain. A total of 10 surgical plans were developed from the CT scan on the Medtronic Stealth™ Station (Medtronic, Inc. Minneapolis, MN, USA) to plan the target and trajectory. The error (3D Euclidean distance from the distal end of the test stylet to the intended target) between the actual and planned electrode position was calculated using the post-operative CT image.

### System mathematics

Mathematical formulas were developed for the transformations achieved by the new adapters using both traditional transformation matrices and geometric algebra. As previously described [10], the primary targeting area of the stereotactic system can be defined as a cube (100 × 110 × 70 mm) where the work envelope is defined. The main surgical targets are within this work envelope, which can be thought of as a sphere whose center may be moved to any target within the work envelope. With this structure, the stereotactic system can be defined to possess 6 degrees of freedom, the X, Y, Z, collar angle, arc angle, and radial distance. The X is the frame’s lateral/medial movement, Y is the anterior/posterior movement, and Z is the superior/inferior movement. These three linear degrees of freedom and the platform placement are in a constant relationship with the work envelope, and the sphere’s center can be linearly moved to any coordinates within the work envelope. This makes it possible to target any point with a probe directed normal to the surface of the sphere. The stereotactic system uses the center of arc principle [10] where the arc and collar angles are oriented along the X–Z and Y–Z planes, respectively, to add two angular degrees of freedom about the center of the sphere. Finally, the last degree of freedom is the radial distance, which is measured by the length of the probe along the trajectory. This distance is set at 150 mm and is a fixed number in the present system.

### System mathematics for adapters—transformation matrices

To determine how usage of each of the adapters would impact the frame’s work envelope coordinate configuration, the Skull Anchor Key was first applied without any adapters, and a CT scan was taken to establish a baseline coordinate system and initial plan values (X, Y, Z, Arc, Collar) using the right superior posterior coordinate as the origin (*x* = 0, *y* = 0, *z* = 0). The initial target and trajectory plan were converted to a target point and cranial entry point, and transformation functions were applied to each. The calculated entry point (X_e_, Y_e,_ Z_e_) was derived using basic trigonometric functions in a three-dimensional space. Starting from the initial target coordinates, the arc and collar angles provided the angles to be used in the equations, and an arbitrary distance of 100 mm was used as the span between target and entry. The resulting equations for the calculated entry point were as follows [23, 24]:

$$\begin{gathered} x_{e} = x_{t} - \rho \cos \theta = x_{t} - 100 \cos \left( {Arc} \right) \hfill \\ y_{e} = y_{t} + \rho \sin \emptyset \cos \theta = y_{t} + 100\sin \left( {Collar} \right) \cos \left( {Arc} \right) \hfill \\ z_{e} = z_{t} - \rho \sin \emptyset \sin \theta = z_{t} - 100\sin \left( {Collar} \right)\sin \left( {Arc} \right) \hfill \\ \end{gathered}$$

In the above equations, $\theta$ is the arc angle and $\varnothing$ is the collar angle. To determine the effect of each adapter on the work envelope, 3-dimensional transformations were applied to both the target point (X_t_, Y_t_, Z_t_) and the entry point (X_e_, Y_e_, Z_e_) based on each individual adapter’s geometry. These physical transformations, such as rotation or translation performed by the adapter, establish in the new coordinate system the new position of each point in step operations. First,rotations about the Z- and X-Axes are applied to (R_x_ (q)) and (R_z_ (q)), respectively Subsequently, a translation to adjust to the correct superior posterior coordinate about the origin is applied. Each rotation uses a standard 3D rotation matrix multiplication, and an example of Z-Axis rotation is provided below. The values used for the rotational and translational adjustments are unique to each adapter and derived using measurements in Solidworks™.

Z-Axis Rotation Matrix Equations:

$$\left[ \begin{gathered} x^{\prime} \hfill \\ y^{\prime} \hfill \\ z^{\prime} \hfill \\ \end{gathered} \right] = \left[ {\begin{array}{*{20}c} {\cos \left( \theta \right)} & {\sin \left( \theta \right)} & 0 \\ { - \sin \left( \theta \right)} & {\cos \left( \theta \right)} & 0 \\ 0 & 0 & 1 \\ \end{array} } \right] \, \times \, \left[ \begin{gathered} x \hfill \\ y \hfill \\ z \hfill \\ \end{gathered} \right]$$

The Prime values are the new coordinates and *θ* is the amount that the *x* axis is rotated towards the *y* axis (counterclockwise). After transforming the target and entry points to the new coordinate system X¢_t_, Y¢_t_, Z¢_t_ and X¢_e_, Y¢_e_, Z¢_e_, the inverse methods to those described above are used to convert these transformed target and entry points back to the (X¢, Y¢, Z¢, Arc¢, Collar¢) values for use with the positioner on the stereotactic frame.

### System mathematics for adapters—geometric algebra

**Fig. 2:**
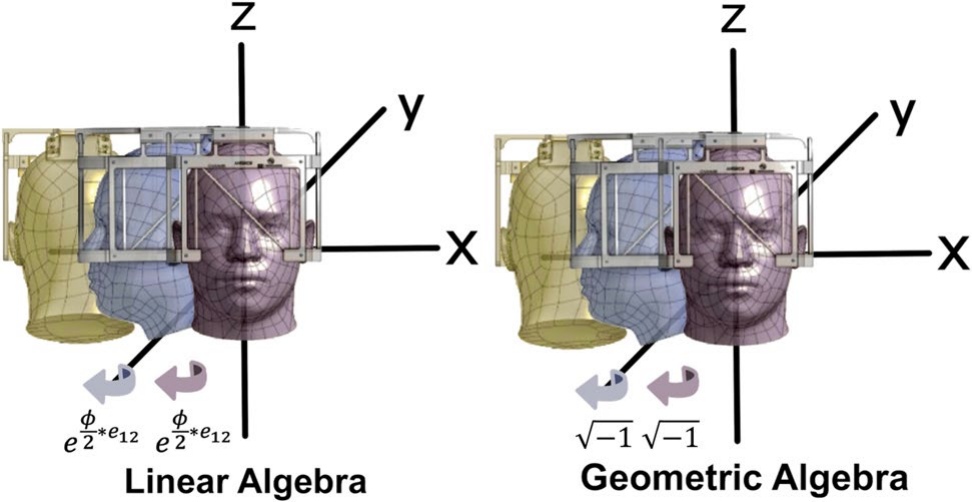
Illustration of geometric algebra. For each multiplication of $\sqrt{-1}$, a 90° rotation occurs. After two rotations, the original figure has been rotated 180°, and therefore multiplied by − 1. In general, to perform multiple rotations, sequentially, one can multiply the associated rotors in order, from left to right

Rotation of the stereotactic system using geometric algebra was achieved using custom-written software. Rotation in geometric algebra is defined by rotors [21, 25]. If the rotor is given by R, then the equation for rotation of any vector v is given by v’ = R^~^vR, where R^~^ is the inverse of R. Because the adapters transform the coordinate system with each rotation, the code for representing the rotor must allow for flexible coordinate bases. In this case, this was handled by representing each rotation sequentially from the perspective of the frame (rather than the perspective of a fixed coordinate plane), and then multiplying the corresponding rotors together in the proper order. Each rotor is defined by the plane of rotation. For example, the rotor describing the rotation that is equivalent to rotating about the Z-axis in Cartesian space is given by:

$${R}_{z}={e}^{\phi *{e}_{12}}$$

where R_z_ is the rotation about the z-axis, $\phi$ is twice the angle of rotation, and e_12_ is the bivector representing the XY Cartesian plane (Fig. 2). In geometric algebra, for each multiplication of $\sqrt{-1},$ a 90° rotation occurs, and after two rotations (180°), it would be multiplied by −1. In general, to perform multiple rotations sequentially, one can multiply the associated rotors in order, from left to right. The rotor equations were developed in Python, and rotations were achieved by multiplying these rotors together in the same order as the rotations described by the adapters.

## Results

### Design of the adapters

**Fig. 3:**
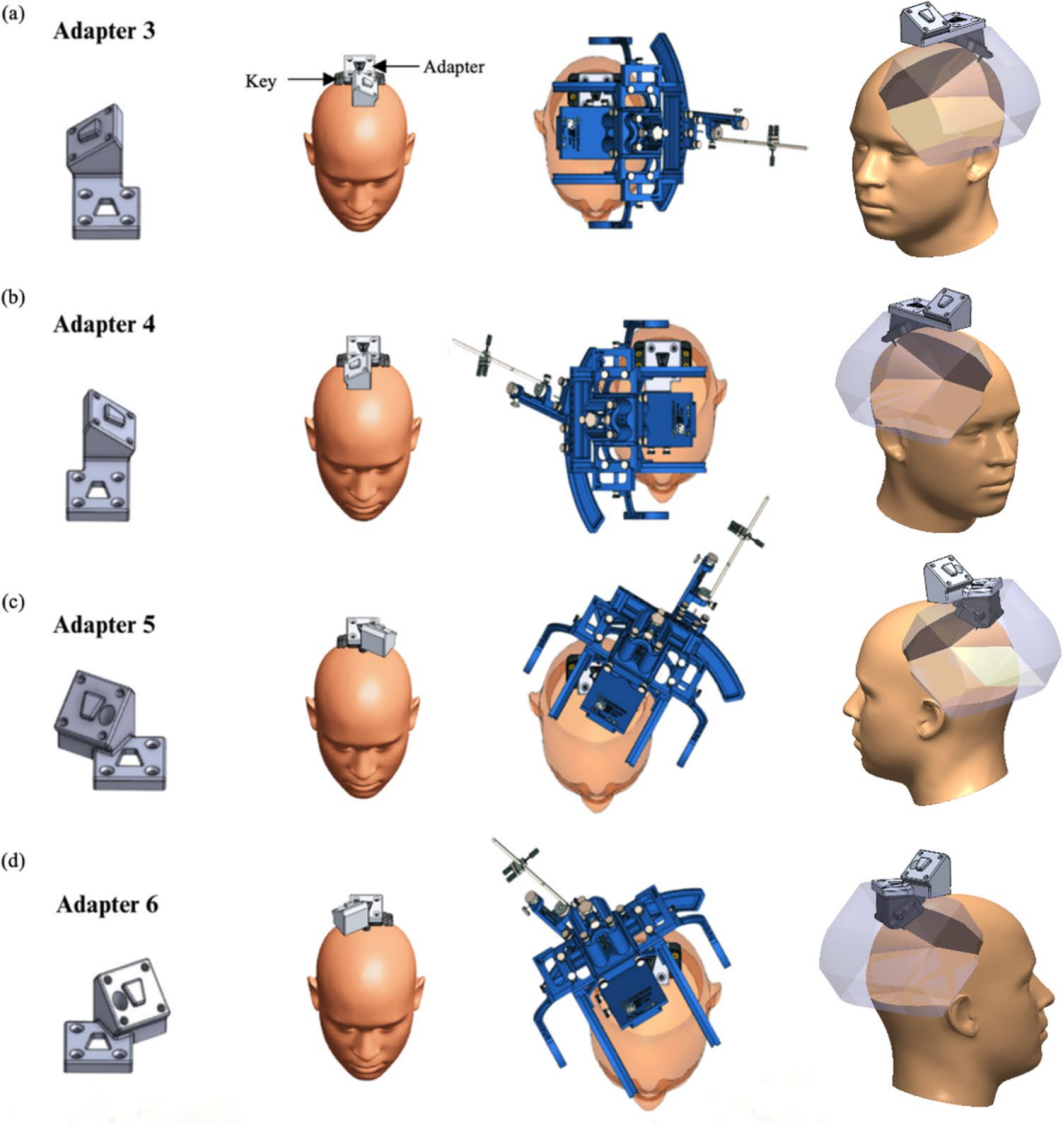
3-Dimensional models of adapters 3, 4, 5, and 6 and their respective orientation on the head and work envelopes. **a** Adapter 3 incorporated a 270° change in rotation about the Z-axis and targets the left side of the head, enabling left lateral-to-medial trajectories. **b** Adapter 4 incorporated a 90° change in rotation about the Z-axis and targets the right side of the head, enabling right lateral-to-medial trajectories. **c** Adapter 5 incorporated a 210° change around the Z-axis that enables left occipital targets using a posterior-to-anterior trajectory. **d** Adapter 6 incorporated a 150° change that enables right occipital targets using a posterior-to-anterior trajectory. Views (**a**–**d**) illustrate the adapter alone, the adapter in use with and without the stereotactic frame and the work envelope enabled by each adapter

**Fig. 4:**
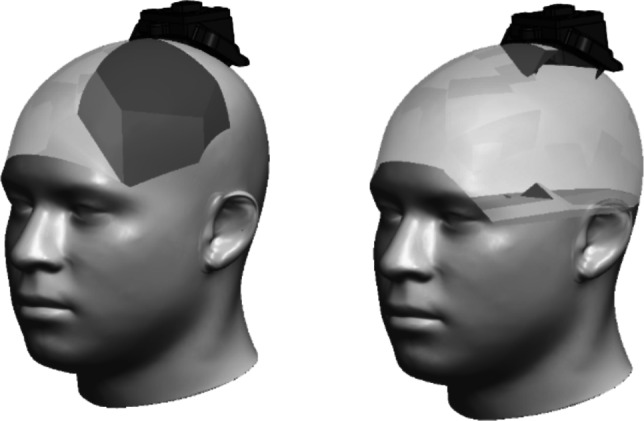
Left, the standard work envelope of the frame; right, the combines work envelope available by utilizing all adapters (entire cranial cavity)

A series of adapters was developed to achieve both rotational and translational change to the stereotactic frame’s work envelope. The adapters were designed to be compatible with the existing “Skull Anchor Key” and frame’s arc-centered delivery system, while maintaining a re-attachable device interface (Fig. 3) by rotations about the Z and X axes that alter the frame’s work envelope and trajectories. With all the adapters, the X and Z-axes of rotation have been altered to allow the original work envelope to be expanded to the entire head. Adapter 3 permits the frame to approach the craniium from the left temporal side (Fig. 3a), whereas Adapter 4 authorizes the approach from the right temporal side (Fig. 3b). Adapter 5enables the approach from the left occipital side (Fig. 3c), and Adapter 6 accommodates the approach from the right occipital side (Fig. 3d). Figure 4 elucidates the original work envelope of the stereotactic frame (left) and expanded available work envelope via the adapters (right).

### Mechanical accuracy testing with ground truth fixture

To ensure the accuracy of the eight manufactured adapters, both rotational matrix transformations and geometric algebra were used to compare targeting of specific test points within the expanded work envelope against known three dimensional points in space. For each adapter, the pre-adapter Cartesian coordinates were converted to post-adapter Cartesian coordinates using transformation matrices and geometric algebra (Table 2). The frame with each respective adapter and a 150 mm targeting probe was secured to the targeting platform and the frame was adjusted to target the known points of the testing fixture. The 3D Euclidean error was then calculated by comparing the X, Y, Z settings from the frame to the converted Cartesian coordinates. The present mechanical error average was 1.75 ± 0.09 mm (*n* = 20 targets).

**Table 2:** Example of one point of interest within the original Cartesian coordinate plane and the changes to the points achieved by each of the 8 adapters

Adapter	X	Y	Z	Collar	Arc
*Initial Plan*					
1	92.7	113.9	87.5	182.3	84.4
*Revised Plan for*					
1	107.3	56.3	143.9	37.7	95.6
2	107.3	75.9	145.8	57.7	95.6
3	91.1	88.1	105.3	54.3	173.9
4	108.9	100.5	97.6	9.7	6.1
5	99.5	95.6	109.9	36.7	135.6
6	106.2	82.3	121.0	37.5	65.6
7	102.3	76.5	125.9	37.0	130.6
8	109.6	82.9	120.6	37.4	60.6

All numbers described in the table are in mm. Adapters 6 and 8 would enable targeting of the initial point due to range of the frame [10]. Identical results were obtained using both geometrical algebra and transformation matrices

### Mock surgical testing

**Fig. 5:**
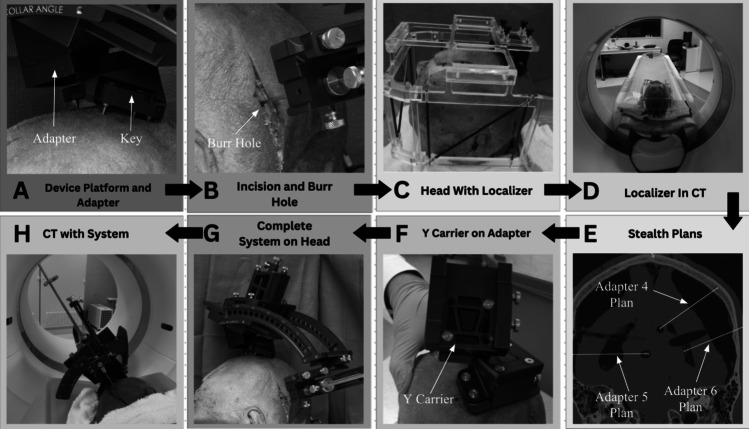
Human cadaver procedure shown in images. **A** Device platform placement on a cadaver specimen and attachment of an adapter on the platform. **B** Precise burr hole incision. **C** Localizer installed on the device platform. **D** Localizer with cadaver head placed in CT to gain a reference Cartesian coordinate plane. **E** Ten different plans within the CT Image made on Medtronic™ Stealth Station (Medtronic, Inc. Minneapolis, MN, USA) to test accuracy. **F** Base setup of an adapter screwed onto a device platform along with the Y-carrier screwed onto the adapter. **G** Complete setup of the stereotactic system with an adapter on the cadaver head. **H** CT scans of each different setup to determine final position to compare with original values of the 10 plans and measure accuracy

**Fig. 6:**
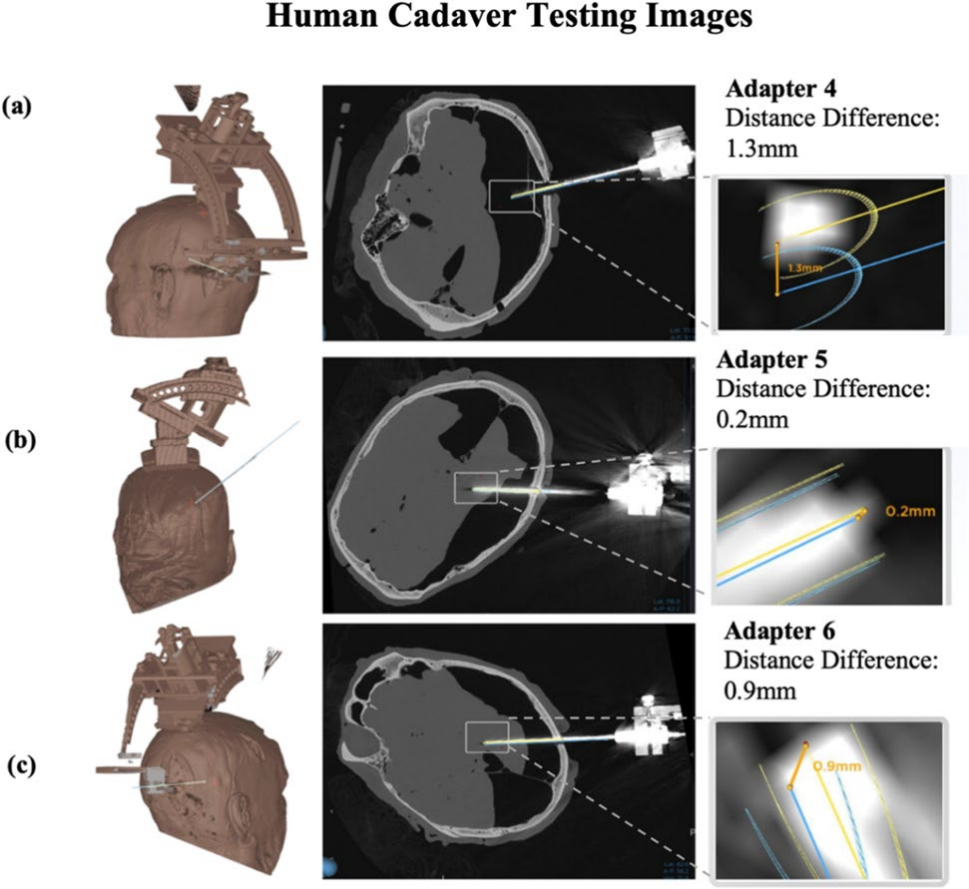
CT Images extrapolated from Medtronic™ Stealth Station. **a** CT image of adapter 4 shown in a 3-dimensional and axial view. The blue plan is the expected implantation location and trajectory of the targeting probe. The yellow line is the actual implantation location and trajectory. The magnified portion indicates the distance measured between the yellow and blue line indicating its accuracy. **b** CT image of adapter 5 shown also in a 3-dimensional and trajectory view. Same as adapter 4, the magnified portion shows the difference in distance indicating the accuracy. **c** CT image of adapter 6 is shown as a 3-dimensional and trajectory view. The accuracy can also be shown in the magnified portion

Next, mock-surgical testing was performed on one cadaveric specimen with three adapters (adapters 4, 5, and 6) for 10 different trajectories. The “Skull Anchor Key” was placed onto the head along with a localizer and obtained an ultra-high resolution CT scan (Fig. 5A, C, D). Ten different surgical plans were created and implemented with the appropriate adapter conversions (Fig. 5E). The appropriate adapter was then attached to the “Skull Anchor Key” and fixed to the skull. The stereotactic frame was then attached to the adaptor and positioned with the converted surgical trajectory coordinates (Fig. 5F, G). An appropriate burr hole was made that was in line with the target trajectory. The stylet was then inserted to the target (Fig. 5B). A post implant CT scan was obtained to calculate the 3D Euclidean distance error (Fig. 5H and Fig. 6). The surgical targeting error was 1.18 ± 0.28 mm (*n* = 10 implantations).

## Discussion

Here, a novel skull mounted adapter system that uses conventional and geometric algebra in conjunction with hardware modifications to a novel stereotactic frame has been developed and demonstrated to expand the work envelope to the entire cranial cavity. This capability should permit further clinical applications that use stereotactic navigation beyond that of DBS targeting. A system of translational and rotational adapters with the principle of geometric algebra allowed total brain navigation capabilities by the stereotactic system. A set of adapters were made that fit between the “Skull Anchor Key” and the stereotactic frame, allowing for both rotation and translation of the work envelope. In addition, the development of mathematical formulas for the transformation matrix allowed new coordinate determination for each of the adapters, using both traditional transformation matrices and geometric algebra. Finally, the system’s mechanical and image-guided accuracies when targeting a ground truth fixture (1.75 ± 0.09 mm, *n* = 20 target coordinates), and in a cadaver head (Fig. 6, 1.18 ± 0.28 mm, *n* = 10 implants), were both under the acceptable 2 mm error limit for targeting brain sites. The mechanical accuracy error was higher than the surgical targeting error because the surgical targeting error is comprised of mechanical error alongside imaging accuracy and additional error. In this instance, the additional error compensated for the mechanical error because they were superimposed in different directions.

### Mathematical transformation using geometric algebra

Both the traditional transformational matrix algebra and geometric algebra were found to provide the same coordinates. However, the mathematics of geometric algebra was much simpler to implement within software. Geometric algebra is a branch of mathematics that incorporates vector geometry to represent rotations with relatively fewer mathematical terms compared to algebraic representations. By using scalars, vectors, cross products, bivectors, and trivectors, rotation within a geometric plane can be represented with a single rotor, which additionally provides a concrete definition of the abstract notion of complex numbers. Indeed, geometric algebra is derived from the idea that complex numbers can be used to represent rotations. Briefly stated, to achieve rotation of a vector by 180° about any arbitrary axis of rotation, the vector is multiplied by −1 along that axis. This is equivalent to multiplying the vector by *i*^2^, and, indeed, multiplying a vector by *i* achieves a 90° counter-clockwise rotation [26]. This concept, as shown in Fig. 2, is crucial to understanding rotations through a combination of geometry and algebra and forms the basis of what would later become geometric algebra. Overall, this method of understanding rotations lends itself to a mathematical model that represents rotations with significantly fewer terms than linear rotation matrices [26].

### Comparison with other methods of representing rotations

The notion of using planes and rotors within geometric algebra to express vector rotation confers several advantages over other mathematical treatments. First, it works equally well in 2 and 3 dimensions (whereas the notion of an axis of rotation does not exist in 2-dimensions). Second, it is fundamentally easier to think about rotations about the plane “in front of the viewer” rather than about an axis orthogonal to the viewer. Three-dimensional rotation matrices are computationally efficient, but are cumbersome to interpret and record, especially when describing multiple sequential transformations. Furthermore, it is inconvenient to convert from rotation matrices to other representations.

Other ways to describe rotation transforms are Euler angles and quaternions. Euler angles are described in terms of sequential rotations about axes attached to an object. It is a suitable representation for this purpose. However, as a system that uses 3 variables to describe rotations in 3-dimensional space, it has potential for introducing singularities (gimbal lock) [27]. In addition, describing two consecutive rotations using Euler angles also leads to cumbersome mathematics that are best avoided in a clinical environment, as the rotations do not simply multiply. Quaternions have a 1:1 correspondence with the bivector algebra of geometric algebra [24]. Both systems are equivalent, but the advantage of geometric algebra is that all rotations are geometrically visible, rather than being consigned to the 4-basis quaternion system. Moreover, applying geometric algebra to a problem will include a variety of representations, including quaternions, allowing one to interconvert between several treatments. Overall, geometric algebra provides a more versatile method for detailing 3-dimensional rotations compared to other treatments, which is ideal for the application of stereotactic surgical systems in the operating room.

Furthermore, the precise targeting and expansion of the work envelope offered by the presented technology would be beneficial for biopsies and other neurosurgical indications in the cranium due to the volume of the procedures performed. In the US alone, 700,000 biopsies are performed per year [29]. Additionally, the proposed work would be more favorable to use over other conventional stereotactic frames due to its skull fixation mechanism. Conventional stereotactic frames utilize the base frame and affix it to the patient, where the base frame surrounds the patient’s head and obstructs some access points to the cranium, such as the occipital or temporal lobe for a biopsy. Because the midline/motor cortex is not the target of conventional neurosurgical procedures, it is subsequently avoided by neurosurgeons. The proposed adapter is designed to be fixed at the midline/motor cortex and therefore, it automatically blocks the system from accessing the midline/motor cortex andit allows other neurosurgical procedures to be operated without obstruction by a base frame. Ultimately, the goal of the presented technology is to expedite and make complex neurosurgical procedures more accessible.

### Limitations, future directions, and conclusion

All measurements including ground truth testing fixture and cadaver accuracy testing were completed with 3D-printed adapters. Due to the nature of 3D printing, some inaccuracy is possible [28]. However, overall dimension difference from 3D CAD and actual printed adapters was 0.15 mm and mechanical accuracy tests with 3D printed adapters showed 0.4 mm which are within acceptable error range of 2 mm in neurological surgery. In addition, new adapters may be machined out of aluminum, which would minimize production variance-based error. The successful usage of the Stealth system was dependent on human input, and therefore human error may be introduced. Incorporation of conversion software into the Stealth system would mitigate human error and increase overall efficiency. The present study has demonstrated the proof of concept that by using specialized custom adapters, the stereotactic system can now expand its work envelope to the whole cranium. Thus, it is our intention to utilize this new capability in other neurosurgical procedures beyond DBS. Thus, it can be envisioned that the stereotactic system may be employed to perform additional stereotactic surgeries, such as stereo-EEG lead placement, stereotactic brain biopsy, laser interstitial thermal therapy, external ventricular drain placement, and ventriculoperitoneal shunt placement.

## Conclusion

This study introduces a novel skull-mounted adapter system that uses geometric algebra and hardware modifications to expand the stereotactic frame’s work envelope to the entire cranial cavity. The system allows rotational and translational navigation for enhanced precision in neurosurgery, with mechanical and image-guided accuracies within acceptable error limits. Geometric algebra simplifies the representation of 3D rotations, offering advantages over traditional methods like Euler angles and quaternions. The system enables access to previously obstructed areas, improving procedures such as biopsies. Future improvements include using machined adapters and software integration to reduce human error and enhance surgical efficiency.

## Data Availability

All data associated with this study are present in the paper or the supplementary materials. All data and materials requests for reproducibility studies, data validation, and research use will be reviewed by KL.
